# Co-design and implementation research: challenges and solutions for ethics committees

**DOI:** 10.1186/s12910-015-0072-2

**Published:** 2015-11-16

**Authors:** Felicity Goodyear-Smith, Claire Jackson, Trisha Greenhalgh

**Affiliations:** Department of General Practice & Primary Health Care, University of Auckland, PB 920919 Auckland, New Zealand; Centre for Primary Care Reform Research Excellence, School of Medicine, University of Queensland, Brisbane, Australia; Nuffield Department of Primary Care Health Sciences, University of Oxford, Oxford, UK

**Keywords:** Co-design, Community-based participatory research, Implementation research, Ethics committee, Intervention studies

## Abstract

**Background:**

Implementation science research, especially when using participatory and co-design approaches, raises unique challenges for research ethics committees. Such challenges may be poorly addressed by approval and governance mechanisms that were developed for more traditional research approaches such as randomised controlled trials.

**Discussion:**

Implementation science commonly involves the partnership of researchers and stakeholders, attempting to understand and encourage uptake of completed or piloted research. A co-creation approach involves collaboration between researchers and end users from the onset, in question framing, research design and delivery, and influencing strategy, with implementation and broader dissemination strategies part of its design from gestation. A defining feature of co-creation is its emergent and adaptive nature, making detailed pre-specification of interventions and outcome measures impossible. This methodology sits oddly with ethics committee protocols that require precise pre-definition of interventions, mode of delivery, outcome measurements, and the role of study participants. But the strict (and, some would say, inflexible) requirements of ethics committees were developed for a purpose – to protect participants from harm and help ensure the rigour and transparency of studies. We propose some guiding principles to help square this circle. First, ethics committees should acknowledge and celebrate the diversity of research approaches, both formally (through training) and informally (by promoting debate and discussion); without active support, their members may not understand or value participatory designs. Second, ground rules should be established for co-design applications (e.g. how to judge when ‘consultation’ or ‘engagement’ becomes research) and communicated to committee members and stakeholders. Third, the benefits of power-sharing should be recognised and credit given to measures likely to support this important goal, especially in research with vulnerable communities. Co-design is considered best practice, for example, in research involving indigenous peoples in New Zealand, Australia and Canada.

## Background

Findings from clinical trials, even when disseminated widely in journals and conference presentations and incorporated into clinical practice guidelines, do not always change clinical practice or produce patient-relevant impacts [1]. One reason for this is that interventions which fit closely to context in one setting (e.g. are acceptable, operationally feasible, affordable and culturally congruent) may be a poor fit in another setting.

The question of ‘fit’ is the basis for the emerging discipline of implementation science – research that addresses the application of interventions in ‘real world’ settings with a view to understanding what works, how and why, in specific contexts, and testing approaches to improve their implementation and effective uptake [2]. Here, the focus is on knowledge production and translation (rather than dissemination of findings), exploring such issues as factors influencing implementation, the results of implementation, and how to scale up an intervention to achieve wider uptake and sustainability.

In conventional clinical trials, different contexts are generally controlled or adjusted for, and populations made as homogenous as possible, with atypical settings and outliers excluded. Protocols are prescriptive and standardised, with the aim to reduce ‘noise’ and create the circumstances for ideal implementation. In contrast, implementation research embraces heterogeneity, addressing complex system integration, and developing an adaptive framework to enable context-sensitive scale-up that is equitable for different populations [3]. Successful implementation involves ongoing adaption to a changing context and feedback processes for longer term spin-offs, transposition and sustainability. Rather than logic models (linking inputs to processes and outputs in a more or less linear way) and point estimates of effect, implementation research uses theory of change, examining natural diversity and the interaction of the intervention in context to produce actionable knowledge. It often uses mixed (quantitative and qualitative) methodologies, and analyses in terms of ‘intention to reach’, rather than ‘intention to treat’, for equitable population health impact [4].

One approach to implementation research involves developing democratic partnerships between researchers and community stakeholders with a view to involving end-users in the design of research, promoting their understanding and capacity, and encouraging uptake of findings [5]. This may involve iterative processes of reflection and action, ‘carried out with and by local people rather than on them’ [6]. Known as participatory action research, this methodology seeks to empower participants to tailor an intervention to suit their own contexts. Interventions need to have contextual fit, to be responsive to the community and the participants they serve [5]. Stakeholders may be the potential recipients of the intervention, or responsible for implementing it, including patients, health care providers, managers and policy-makers. Ideally, collaboration occurs between researchers and end users from the onset – in question framing, research design and delivery, and influencing strategy. This co-creation approach places end-user value at its very heart, with implementation and broader dissemination strategies part of its design from gestation. It also generally seeks to establish an ongoing, collaborative approach between researchers and end users, which is long-term rather than programme or project-specific; and which builds synergistically as partners build trust, work together and learn from successive projects [5, 7].

Ramaswamy et al., in their ‘Cocreation Paradigm’, describe six steps toward a culture of operational co-creation:

1. Identify the key stakeholders and promote their willingness to engage
2. Set up platforms purposefully designed to engage more co-creatively
3. Identify and support new champions
4. Expand the circle of stakeholders and joint value creation opportunities
5. Deepen the impact and enable the spread of more win-more, win-more strategies
6. Engage stakeholders to expand benefits for all [8].

Examples of the success of this approach in ‘locking down’ research uptake into the dynamic and time-constrained environment of community-based healthcare are accumulating [9–13]. However, because collaborative partnerships inevitably bring logistical complexities as well as competing values and conflicts of interest, challenges to research governance, flexibility and timeframes are common [9, 10, 14–16].

One particular challenge in co-creation research, and the focus of the remainder of this paper, relates to the traditional ethics approval processes.

## Discussion

### Challenges in gaining ethics committee approval in co-creation research

Research involving human participants puts people at risk, largely for the benefit of the researchers [17]. The current system of research ethics in biomedicine emerged partly as a response to international scandals such as the infamous experiments on prisoners under the Nazi regime and the Tuskeegee syphilis study (in which life-saving treatment was withheld from poor Black Americans, who had no knowledge of the research, for more than 30 years) [18]. Building on the landmark Nuremberg Code [19] the Declaration of Helsinki [20] and the International Ethical Guidelines for Biomedical Research Involving Human Subjects, [21] medical research has become increasingly regulated in the past five decades. Formal codes of practice are now the norm, with the goal of ensuring that the methodology is rigorous; that potential harm to participants is minimised; that people know exactly what they are signing up to; and that the potential benefits of study outcomes outweigh possible risks of safety or confidentiality.

To that end, current clinical ethics and governance procedures are oriented to confirming that research interventions (drugs, procedures, educational or behavioural measures) are carefully defined and explained to potential participants before they agree to join the study; that consent procedures are non-coercive; that people receive no more or less than the intervention(s) stipulated in the protocol; that responses to that intervention are systematically monitored; that the welfare and choices of participants remain paramount (even if this necessitates withdrawing people from a study or compromising on what data are collected); and that a clear trail of accountability can be demonstrated should harm occur. Such procedures have been criticised for being bureaucratic, inefficient and inconsistent, [22] but there is broad agreement that a stringent and transparent system of governance is preferable to a repeat of past scandals [23].

By their nature, intervention studies (offering a drug, procedure or complex intervention with the aim of preventing, diagnosing or managing a condition and then assessing its effectiveness and safety) carry greater risk to participants than observational studies (in which no active manoeuvre is offered beyond usual care). Ethics committees rightly view themselves as responsible for what interventions are offered, to whom, and how. Furthermore, as much clinical research has become centred on randomised controlled trials (many of which are sponsored by industry), research governance has evolved to ensure that clinical trials are registered at the outset to prevent selective censorship of negative results [24] and to reduce protocol deviations (e.g. alteration of drug dose or scheduling, change of primary endpoint or length of follow-up) designed to influence the outcome [25].

Increasingly, therefore, the role of research ethics committees (institutional review boards in USA) is to carefully check study protocols and supporting documentation in advance of an intervention study and then ensure that these are strictly adhered to for its duration. For complex interventions, pre-specified information is likely to include participants’ anticipated time commitment, interview schedules, questionnaires and so on. This rational and (some would say) technocratic approach means that flexibility, adaptation to context and the very essence of co-creation is systematically ‘designed out’.

Pre-specification of the study protocol and other documents is a quality feature in randomised trials. But when the focus of research is the achievement of intervention-system fit (as in implementation science) and/or the emergent co-creation of research through multi-stakeholder partnerships, rigid pre-specification is at best ironic and at worst a recipe for failure, since by definition, the definitive intervention and its application is not pre-determined. Rather, the research question, the nature and delivery of the intervention and how its impact is measured, must be co-determined by researchers and other stakeholders – usually over weeks or months and in parallel with establishing programme governance, developing research capacity in community partners, building trust and working through conflict. These are ongoing and mutually reinforcing processes, not one-off procedures that can be ticked off as having been ‘achieved’.

Because of the emergent nature of the co-creation process and the crucial importance of relationships and partnership synergy, compromises may need to be made with respect to methodology to facilitate impact. But this emergent methodology creates unique challenges to ethics committees, whose protocols, as noted above, tend to require precise definition of the intervention, its mode of delivery, outcome measurements, and the role of participants in the study.

### Some principles for handling ethics committee applications in implementation research involving co-creation elements

The need (on the one hand) to co-create and adapt interventions for the purposes of maximising fit and (on the other hand) to pre-specify them for the purposes of ethical approval creates a tension for which there is no simple or formulaic solution. Furthermore, it is important, whilst acknowledging the need for flexibility in some studies, not to create loopholes that will allow partisan interests to ‘move the goalposts’ in more conventional research designs.

Furthermore, ethics committees aim to protect the autonomy and rights of individuals participating in clinical research, but individually-based ethical frameworks are not necessarily of benefit to the community at large, who are collectively involved in the research process [26]. With co-creation design, there is a move from protection of individual participants to the development of a relationship between researchers and community partners which is mutually advantageous [27]. The participant is not someone on whom research is ‘done’, but is actively engaged in designing and implementing the research process.

Banks et al., writing about community-based participatory research (CBPR, a type of co-creation research linked to community development and the quest for social justice), point out that whereas conventional research ethics is principle-based and focused primarily on ensuring rule-following to meet regulatory requirements, CBPR is value-based and focused on relationships and the distribution of power between ‘researchers’ and ‘researched’ (and acknowledging that the boundaries between these groups may be blurred) [28].

With these caveats in mind, and assuming that co-creation approaches will continue to be assessed by existing systems of research governance, we offer four principles to guide ethics committees when considering applications.

First, ethics committees should acknowledge and celebrate the diversity of research – both formally (by ensuring that ethics committee members receive broad training in the full range of research methods, study designs and the rationale for these) and informally (by encouraging discussion and debate). The make-up of research ethics committees may be very diverse, with individual members who may be selected for their professional expertise in different fields (for example nursing, law, ethics) and/or for their ‘lay’ contribution – but these members may have little or no formal training in research methodologies [29]. Even those well versed in laboratory science or randomised trials may have little knowledge or understanding of the characteristics of high-quality implementation and/or co-design research. Researchers can play their part in educating committee members on the nature of such research, and why a co-creation approach may increase the likelihood of impact and reduce harm to participants. Other commentators have similarly highlighted the importance of educating ethics committees in this regard [30]. Approaches may include researchers explicitly mentioning these issues in their application; supplying a key paper outlining this type of research as supplementary material; or seeking the opportunity to meet with the committee and explain co-design in the context of their proposal.

Second, researchers and other stakeholders in the co-creation process should work with research ethics committees, perhaps at national level, to establish some ground rules for participatory research applications and communicate these to all parties. For example, preliminary consultation with potential beneficiaries (‘patients and carers’) is already defined by most ethics committees as a necessary step before an application can be submitted. While a Code of Research Ethics has previously been developed for participatory research, [31] this was not intended to be imposed on all research; rather to be individually developed for different projects [32]. Establishing partnership governance and outlining the core components of a planned intervention can and should begin before the ethics application is finalised. There are likely to be some components that are integral aspects of the intervention, not amenable to change, and other aspects such as the setting or application that can be co-created. Hawe et al’s distinction between the theoretical ‘core’ of a complex intervention, which must be kept constant, and its local application, which can and should be flexible across sites and settings, may be a useful conceptual framework [33]. It will often (though perhaps not always) be possible to make explicit the elements open to modification, and stipulate the nature of the facilitation process through which this will occur.

Third, research ethics committees should acknowledge the benefits of power-sharing in the co-design process and give credit to measures that support this goal; researchers in turn should make such measures explicit in their ethics applications. The reciprocity of co-creation serves to diminish participant risk. Researchers and stakeholders work together, and this power-sharing – to the extent that it is effective – will serve to reduce inequality and empower vulnerable communities. Co-design, for example, is considered best practice in research involving indigenous peoples (some of whom are especially vulnerable and/or have been exploited by researchers in the past [34]). In New Zealand, best practice is methodology that empowers indigenous Māori to take a governance role in the ‘planning, development and execution of research as well as monitoring the project through its life cycle’ [35]. In Australia, it is recognised that ‘indigenous communities and individuals have a right to be involved in any research project focused upon them and their culture’ and researchers are encouraged to include Traditional Owners, custodians, Elders, and community members to be involved in the research as collaborators, advisers or assistants [36]. Similarly the Canadian Panel of Ethics directs that research involving Aboriginal peoples in Canada, including Indian (First Nations), Inuit and Métis peoples, should be premised on respectful relationships, and encourages collaboration and engagement between researchers and participants [37].

Finally, our fourth principle is one of emergence and continuing learning by all parties. The ethics of co-creation are an emerging area on which there is, appropriately, ongoing debate. Other commentators in the recent literature have made suggestions about how ethics committees may consider participatory research. Chen and colleagues, for example, suggest a framework which includes respect for potential and enrolled participants, community partners and research partners [38]. Another study failed to find consensus regarding who represents and speaks for a community [26]. Blake proposes participants only signing consent forms after all interviews or focus groups have been completed, and including preferences regarding anonymity and use of audio recordings [39].

To continue to inform the debate, we suggest that data should be prospectively collected on the types of applications that are received by research ethics committees how issues such as the lack of a definitive intervention and its application have been addressed, and identification of the learning points, and these findings disseminated. Examination of such cases and how they have been resolved will help to move the debate onwards. Collecting and assessing such data could be a role for organisations such as the National Research Ethics Service (now part of the Health Research Authority) in the UK, and the Health and Disability Ethics Committee in New Zealand, which are charged with ensuring that the rights and safety of research participants are protected, and ethical research of potential benefit to science and society is conducted.

## Conclusions

There is no simple answer to the question of how to balance the laudable principle of co-creation with the need for robust and transparent governance of research on human participants. The principles proposed in this paper are preliminary; we invite discussion and recommend further research on the ethical dimension of this rapidly developing field.
